# The Autism‐Spectrum Quotient in Siblings of People With Autism

**DOI:** 10.1002/aur.1651

**Published:** 2016-06-22

**Authors:** Emily Ruzich, Carrie Allison, Paula Smith, Howard Ring, Bonnie Auyeung, Simon Baron‐Cohen

**Affiliations:** ^1^ Cambridge Intellectual and Developmental Disabilities Research Group, Department of Psychiatry, University of CambridgeCambridgeUK; ^2^ Autism Research Centre, Department of Psychiatry, University of CambridgeCambridgeUK; ^3^ NIHR CLAHRC for the East of EnglandCambridgeUK; ^4^ Cambridgeshire and Peterborough NHS Foundation TrustPeterboroughUK; ^5^ CLASS Clinic, Cambridgeshire and Peterborough NHS Foundation TrustPeterboroughUK; ^6^ Psychology DepartmentUniversity of EdinburghEdinburghUK

**Keywords:** autism, autism‐spectrum quotient, autistic traits, siblings, sex differences

## Abstract

This study measures the distribution of autistic traits, using the autism‐spectrum quotient (AQ), in siblings of individuals with autism spectrum conditions (ASC). Total AQ scores, along with AQ subscales, were collected from child, adolescent and adult controls, siblings, and volunteers with ASC using one of the three age‐appropriate versions of the instrument: the AQ (adult self‐report), the AQ‐adolescent and AQ‐child (both parent‐reports). We examined the effect of Group (case, sibling and control) and AQ version (adult, adolescent and adult) on total and subscale scores. In addition, we tested for sex differences in all groups and on all versions. We found that in male and female adults, AQ scores in siblings fell between cases and controls (cases > siblings > controls). In children and adolescents, female siblings also scored higher than control females (female cases > female siblings > female controls), but there was no difference between male siblings and controls (male cases > male siblings = male controls). An investigation of subscale scores revealed that male siblings only differed from controls on the “Communication” subscale (male cases > male siblings > male controls), while female siblings differed from controls on all subscales except “Imagination” (female cases > female siblings > female controls). This study confirms the broader autism phenotype in siblings, and reveals this is modulated by sex and AQ version. ***Autism Res*** *2017, 10: 289–297*. © 2016 The Authors Autism Research published by Wiley Periodicals, Inc. on behalf of International Society for Autism Research.

## Introduction

Although autism is a discrete clinical diagnosis, autistic traits exist along a continuum that extends into the general population. This has been confirmed using behavioral measures of autistic traits such as the autism‐spectrum quotient (AQ), a 50‐item measure for quantifying autistic traits in individuals with average IQ or above [Baron‐Cohen, Wheelwright, Skinner, Martin, & Clubley, 2001]. While other measures of autistic traits have also been developed, the AQ has the advantage of primacy, generalizability and free distribution compared to instruments such as the broad autism phenotype questionnaire (BAPQ) [Hurley, Losh, Parlier, Reznick, & Piven, 2007], the subthreshold autism trait questionnaire (SATQ) [Kanne, Wang, & Christ, 2012], and the social responsiveness scale‐adult self‐report (SRS2‐AS) [Constantino & Cruber, 2012].^1^ The AQ indicates that while most individuals with autism have an AQ score above a cut‐off of either 26 for use in a clinical setting [Woodbury‐Smith, Robinson, Wheelwright, & Baron‐Cohen, 2005] or 32 for use in a general population sample [Baron‐Cohen et al., 2001], there is a continuous distribution of scores across individuals with and without a diagnosis, substantiating earlier claims of a continuous severity gradient of autistic traits [Wing, 1988]. Studies using the AQ have previously shown that autistic traits exist to a lesser degree in individuals without a clinical diagnosis of autism spectrum conditions (ASC) [Ruzich et al., 2015a]. In fact, certain groups within the general population may be predisposed to have higher levels of autistic traits and may be more at risk for ASC. For instance, there are consistent sex differences in traits associated with autism, such that typical males appear significantly more similar to people of both sexes with ASC than do typical females [Baron‐Cohen, 2002; Baron‐Cohen et al., 2014]. In addition, within families, autistic traits have shown heritability, such that some (but not all) parents of children with autism show a subclinical set of characteristics or traits that index familial inheritance and/or genetic liability to autism [Bishop et al., 2004; Hoekstra, Bartels, Verweij, & Boomsma, 2007; Wheelwright, Auyeung, Allison, & Baron‐Cohen, 2010]. Further, a genetically linked broader autism phenotype (BAP) can be described in undiagnosed mothers and fathers of children with ASC [Wheelwright et al., 2010].

Wheelwright et al. [2010] reported that both mothers and fathers of children with ASC have significantly higher AQ scores than parents of children without ASC. That study showed that on four out of the five AQ subscales, parents of children with ASC score significantly higher than parents of children without ASC. This confirmed and extended a smaller study [Bishop et al., 2004]. While both siblings and parents share on average half their genetic makeup with affected individuals, a similar effect on AQ scores has not previously been measured in siblings of individuals with ASC. However, studies have evaluated performance on behavioral and cognitive measures in siblings of individuals with ASC with mixed results. On four different tasks measuring central coherence, fathers but not brothers were shown to have reduced performance compared to controls [Happé, Briskman, & Frith, 2001]. A study of social behavior in siblings using the social responsiveness scale (SRS) found siblings of individuals with ASC to be impaired [Constantino et al., 2006]; however, in a later study using the same measure, an effect on social behavior was only found in fathers and not in mothers or siblings [De La Marche et al., 2012]. In contrast, a recent functional magnetic resonance imaging (fMRI) study showed that siblings of individuals with ASC had a significantly reduced response to happy versus neutral faces in regions of the brain associated with face processing and empathy [Spencer et al., 2011]. The latter study suggests an underlying cortical difference that may not always result in different behavior. For more complete summaries of sibling studies, see Wade, Prime, & Madigan [2015] and Pisula & Ziegart‐Sadowska [2015]. The AQ includes behavioral subscales that extend beyond the social domain and may prove useful in investigating whether the observed trend of a BAP in parents of individuals with autism is also found in siblings.

In the current study, we predicted that individuals with a sibling with ASC would score higher on the AQ than controls, but lower than participants with ASC. We also tested for the effect of AQ version (child, adolescent and adult) on scores for each participant group. The AQ‐child and AQ‐adolescent are rated by parents and are based on the AQ adult version (which is a self‐report measure). While the AQ parent‐report versions are based on the self‐report AQ for adults, there may be differences in how outside observers rate an individual's autistic traits as compared to how an individual perceives him or herself. Previously, this has been demonstrated both behaviorally [Furlano, Kelley, Hall, & Wilson, 2015] and using neuroimaging [Lombardo et al., 2010]. We hypothesized that there would be differences in AQ scores by group according to whether the measure was completed by self‐ or parent‐report. In addition, we examined sex differences in AQ scores within the sibling group, as previous research has identified a small but significant difference in AQ scores between males and females in the general population, a difference that is absent in males and females with ASC [Baron‐Cohen et al., 2014, 2001; Wheelwright et al., 2006].

## Methods

### Instrument

Development of the AQ is described elsewhere [Auyeung, Baron‐Cohen, Wheelwright, & Allison, 2008; Baron‐Cohen, Hoekstra, Knickmeyer, & Wheelwright, 2006; Baron‐Cohen et al., 2001]. The AQ consists of 50 items, divided into five subscales consisting of 10 items each. It assesses domains of cognitive strengths and difficulties related to ASC: communication, social skills, imagination, attention to detail and attention switching. The AQ (adult version) is designed as a self‐report questionnaire, while the adolescent and child versions are completed by a parent or caregiver. The AQ was designed for individuals with average IQ or above [Baron‐Cohen et al., 2001], who comprise at least 50% of the autism spectrum [Baird et al., 2006; (Centers for Disease Control and Prevention [CDC], 2014). It is not suitable for individuals with low IQ, low verbal ability, or language impairment, as it relies on comprehension of each of the 50 questions. Individuals are instructed to respond to each of the 50 items with 1 of 4 responses: “definitely agree,” “slightly agree,” “slightly disagree,” and “definitely disagree.” For the purposes of the current study, responses are scored using a binary system, where an endorsement of the autistic trait (either mildly or strongly) is scored as 1, while the opposite response is scored as 0, leading to a maximum score on the AQ of 50. AQ items are counterbalanced to avoid a response bias, so that half of the “agree” responses and half of the “disagree” responses endorse the autistic trait. The AQ includes questions about both ability and preference.

In addition, we used the AQ‐adolescent and AQ‐child. The AQ‐adolescent [Baron‐Cohen et al., 2006] consists of the same 50 items, subscale categories, question format, and response styles as the adult AQ. However, it differs in that it is a parent – rather than a self‐report, using the term “S/he” rather than “I.” The AQ‐child is, like the AQ‐adolescent, a parent‐report consisting of 50 items [Auyeung et al., 2008]. It was designed to be suitable for children of average IQ aged from 4 to 11 and, while the format of the questionnaire remains the same as the AQ‐adolescent, several of the items were rewritten to better assess autistic traits in this group using age‐appropriate questions. Out of the 50 items, 12 were altered to be age‐appropriate (items 6, 7, 8, 13, 15, 20, 21, 24, 26, 32, 40 and 48). For instance, in item 21, the AQ‐adolescent reads: “S/he does not particularly enjoy reading fiction,” while the AQ‐child reads: “S/he does not particularly enjoy fictional stories.”

### Participants

Three groups of participants were selected: individuals with a clinical diagnosis of ASC (cases), individuals with no personal history of ASC but with a sibling with a diagnosis (siblings), and individuals with no personal or family history of ASC (controls). Within each group, three cohorts were selected: those who had taken the 50‐item AQ self‐report (suitable for adults), the AQ‐Adolescent parent‐report, and the AQ‐Child parent‐report (see Table 1). Given that the AQ is recommended for individuals with average IQ or above, parents were asked during the recruitment and enrolment process about whether their child had any learning or language difficulties. Only participants who reported no impairments were selected for the study.

**Table 1:** Participant Characteristics

	ASC	Siblings	Controls
*AQ*			
Males	897	69	344
Females	897	69	344
Total	1794	138	688
Mean age in years (SD)	35.34 (12.98)	28.22 (12.11)	43.46 (4.98)
*AQ‐adolescent*			
Males	87	46	296
Females	87	46	296
Total	174	92	592
Mean age in years (SD)	13.89 (1.14)	13.71 (1.14)	14.29 (0.97)
*AQ‐child*			
Males	177	133	360
Females	177	133	360
Total	354	266	720
Mean age in years (SD)	7.69 (2.19)	7.96 (2.33)	9.76 (1.38)

#### ASC cases

Participant data from individuals with a formal diagnosis of ASC were collected from the Cambridge autism research database (CARD). Here, volunteers (or their parents/caregivers) can register online (www.autismresearchcentre.com) and provide details about their diagnosis and complete an online version of the AQ. Only individuals who provided full diagnostic information (location, clinical psychologist or psychiatrist and date of diagnosis) and completed the AQ test were included in the analysis.

#### Siblings

Participant data from siblings of individuals with ASC, hereafter referred to as the sibling group, were also collected from CARD. Parents of children and adolescents were recruited and invited to respond on behalf of each of their children, regardless of diagnosis. For adults, volunteers were invited by their brother or sister with ASC via an online form (www.autismresearchcentre.com); a minority of adults from the general population interested in taking part in psychology research also were recruited online at a site seeking nonclinical controls (www.cambridgepsychology.com). For this group, individuals were selected if they had co‐registered with a sibling with ASC or if they had reported having a sibling with ASC, even if the sibling had not registered online. Individuals were excluded if they reported a suspected diagnosis or reported details of a diagnostic assessment for ASC, even if the assessment had not resulted in a formal diagnosis of ASC. While these individuals were siblings of an individual with ASC, they were not the sibling of any of the individuals from the ASC participant group, to ensure the groups were independent.

#### Controls

Participant data from individuals with no personal or family history of ASC were collected from the SCORE cohort [Allison et al., 2007; Auyeung et al., 2009]. Participants were asked to answer the yes/no question, “Have any of your family members been diagnosed with an autism spectrum condition?” Participants were not asked to specify which relations were or were not diagnosed with ASC. In the SCORE study, questionnaires were distributed via schools in the Cambridgeshire area to parents/caregivers and their children (initially recruited aged 5–9 years old; re‐contacted for AQ data when aged 6–11) and were returned to researchers by Freepost envelope. Parents were asked to fill out questionnaires about their own autistic traits and on behalf of their children. Families were later re‐contacted when children were of an appropriate age to fill out the AQ‐adolescent.

#### Processing

Participant data were imported into R [RCoreTeam, 2014] (7618 individuals), cleaned of incomplete AQ tests by removing individuals with null scores or missing data, and exclusions were made if the participant was older or younger than the age range recommended for each test (AQ‐child 4–11; AQ‐adolescent 12–16; AQ 16+) – 1011 excluded. Individuals duplicated between age groups (e.g., an individual whose parent had completed the AQ‐child and then later had completed the AQ‐adolescent) were randomly selected for inclusion in just one of the measures using a process that maximized each group's sample size, and multiple members of a family were removed (e.g., if two siblings of an individual with autism had taken the AQ, only one was included for analysis using an automated and randomized selection procedure) – 450 excluded. Equal numbers of males and females were included via a random selection process. Table 1 describes the available samples for analysis. Given the finding in the literature that there is a small but significant difference in AQ scores between males and females in the general population, and which is absent in males and females with ASC, participant groups comprised equal numbers of males and females. Comparisons are reported separately by sex.

### Procedure

Descriptive statistics for AQ total and subscale scores were examined. The effects of group, sex and version on AQ score were evaluated. Distribution of AQ scores was evaluated for each group; maximum skew value = 1.35 for male child siblings and maximum kurtosis value = 1.25 for female child siblings. As skew and kurtosis did not exceed 2.00 standard deviations from the mean, parametric tests were used. Further, ANOVA and the F‐statistic are robust to violations of assumptions of normality [Donaldson, 1968].

## Results

### Mean AQ Scores

**Figure 1:**
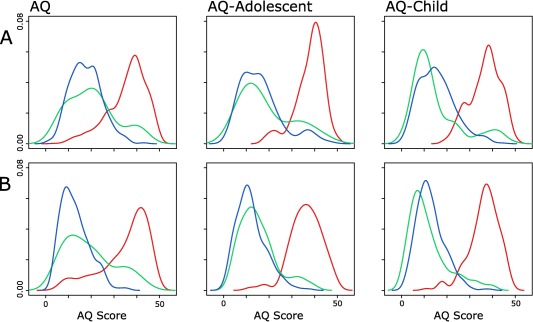
Kernel density estimates for AQ score distributions for (A) males and (B) females. Blue denotes control group, green denotes siblings and red denotes ASC group.

Mean AQ scores and standard deviations for all groups are shown in Table 2. Score distributions as indicated by kernel density plots are shown in Figure 1. This figure also hints at a bimodal distribution of scores in siblings, absent in controls and individuals with ASC. However, detailed investigation of this distribution is beyond the scope of this study (for a complete account of this investigation, see Ruzich et al. [2015b]. Within each group, the effect of age on AQ score was tested, with no significance found in 17 out of 18 comparisons, the exception being within adult males with ASC (*P* < 0.001).

**Table 2:** AQ Descriptive Statistics for Males and Females

		Males		Females	
		Mean (SD)	Range	Mean (SD)	Range
AQ	ASC	35.74 (8.59)	4–50	34.96 (10.61)	3–50
	Sibling	20.42 (10.79)	3–45	19.25 (11.03)	4–44
	Control	17.62 (6.90)	1–43	12.90 (6.25)	3–36
AQ‐adolescent	ASC	37.87 (6.31)	18–50	36.13 (6.84)	12–49
	Sibling	18.70 (11.66)	2–47	15.17 (8.24)	4–39
	Control	16.26 (9.43)	1–48	12.13 (6.54)	0–38
AQ‐child	ASC	36.70 (6.70)	19–50	36.28 (6.56)	11–48
	Sibling	15.08 (11.17)	0–49	11.79 (8.71)	1–40
	Control	15.52 (7.90)	1–44	12.72 (6.10)	1–39

First, to determine if males and females in the sibling group conform to the pattern found in controls where males score significantly higher than females on the AQ, or to the pattern found in individuals with ASC where AQ scores have no sexual dimorphism, a two‐sample *t*‐test was performed between males and females in the sibling group for each version of the AQ. For child siblings, there was a significant difference between males and females; *t*(264) = 2.68, *P* < 0.01. However, no sex difference was found for adolescent or adult siblings in the current sample.

Analysis of variance (ANOVA) examining the effect of participant group (ASC, sibling and control) and AQ version (AQ, AQ‐adolescent and AQ‐child) was carried out on total AQ scores. Males and females were considered separately in an effort to reduce comparisons and maintain power. For males, there was a significant main effect of group, *F*(2) = 1577.21, *P* < 0.001, with Bonferroni post hoc tests revealing that males with ASC had significantly higher scores than male siblings and controls, but that male siblings and controls did not differ. There was also a main effect of version, *F*(2) = 4.09, *P* = 0.02, revealing that parent‐report versions of the AQ return more polarized scores (that is, scores are lower for controls and higher for ASC participants) than does the AQ self‐report. There was also a significant group‐by‐version interaction, *F*(4) = 7.19, *P* < 0.001. To unpick this interaction and to determine if male siblings and controls differed for any of the individual AQ versions (a difference that was not apparent when assessing combined parent‐ and self‐reports), simple comparison t‐tests were performed to compare male siblings and male controls for Child, Adolescent, and Adult versions. For Adult males, siblings scored significantly higher than controls (*P* < 0.01), while for the child and adolescent versions, there were no significant differences between male siblings and controls. Effect sizes were calculated: the effect size for participant group was moderate (*η* ^2^ = 0.51), for the group‐by‐version interaction was small (*η* ^2^ = 0.01), and for AQ version was negligible (*η* ^2^ = 0.003).

For females, an identical analysis was run. There was a significant main effect of participant group, *F*(2) = 2073.26, *P* < 0.001, with Bonferroni post‐hoc tests revealing that females with ASC had significantly higher scores than female siblings, and female siblings had significantly greater scores than female controls. There was no significant main effect of AQ version for females, *P* = 0.65. There was, however, a significant group‐by‐version interaction, *F*(4) = 10.12, *P* < 0.001. Post hoc comparisons were used to unpick this interaction and determine if versions differed for any of the groups: for females with ASC and control females, self‐reports and parent‐reports did not statistically differ, while for female siblings, self‐reports had significantly higher AQ scores than did parent‐reports when taken as a group (*P* < 0.01). For females, effect size for group was moderate (*η* ^2^ = 0.58) and for the interaction was small (*η* ^2^ = 0.02).

### Mean Subscale Scores

Analyses were then run independently for each subscale for each sex individually. Mean scores and standard deviations for AQ subscales are shown in Table 3. Separate ANOVAs were run to examine the effect of version and group on each of the five subscale domains (Table 4).

**Table 3:** AQ Subscale Descriptive Means and Standard Deviations

		Communication	Social skills	Imagination	Attention to detail	Attention switching
*Males*						
AQ	ASC	7.44 (2.41)	7.60 (2.43)	6.01 (2.49)	6.84 (2.12)	7.98 (1.99)
	Sibling	3.41 (2.75)	3.70 (3.17)	3.06 (2.55)	5.01 (2.46)	5.25 (2.68)
	Control	2.60 (1.89)	3.05 (2.53)	3.07 (2.05)	4.69 (2.10)	2.14 (2.14)
AQ‐adolescent	ASC	7.97 (1.85)	7.90 (1.93)	7.36 (2.21)	6.53 (2.06)	8.13 (1.65)
	Sibling	3.20 (3.25)	2.52 (2.71)	2.84 (2.67)	4.67 (2.38)	4.50 (2.85)
	Control	2.62 (2.54)	2.52 (2.71)	2.84 (2.32)	4.30 (2.48)	3.97 (2.57)
AQ‐child	ASC	8.41 (1.60)	7.02 (2.08)	6.38 (2.29)	6.72 (2.30)	8.17 (1.75)
	Sibling	2.91 (2.86)	2.18 (2.73)	2.26 (2.32)	4.05 (2.73)	3.68 (2.71)
	Control	2.66 (2.27)	2.11 (2.21)	2.39 (1.99)	4.73 (2.66)	3.63 (2.24)
*Females*						
AQ	ASC	7.16 (2.84)	7.42 (2.77)	5.67 (2.70)	6.92 (2.22)	8.01 (2.39)
	Sibling	3.29 (2.63)	3.55 (3.18)	2.52 (2.33)	5.01 (2.36)	4.87 (2.68)
	Control	1.70 (1.61)	1.87 (2.04)	1.69 (1.63)	4.61 (2.21)	3.03 (2.05)
AQ‐adolescent	ASC	8.15 (1.85)	7.68 (1.91)	5.95 (2.48)	6.14 (2.19)	8.21 (1.68)
	Sibling	2.37 (2.19)	2.72 (2.59)	2.02 (2.02)	4.00 (2.23)	4.07 (2.23)
	Control	1.73 (1.77)	1.45 (2.05)	1.34 (1.50)	4.46 (2.12)	3.15 (2.10)
AQ‐child	ASC	8.31 (1.72)	7.10 (2.10)	6.14 (2.48)	6.46 (2.13)	8.28 (1.71)
	Sibling	2.37 (2.49)	1.52 (2.12)	1.06 (1.65)	3.87 (2.54)	2.97 (2.46)
	Control	2.02 (1.74)	1.40 (1.73)	1.35 (1.38)	4.77 (2.35)	3.13 (1.87)

**Table 4:** ANOVA Results for Subscales

	Communication	Social skills	Imagination	Attention to detail	Attention switching
*Males*					
Group *F* value (*η* ^2^)	1339.15** (0.49)	1208.45** (0.42)	644.23** (0.31)	282.28** (0.15)	1020.44** (0.40)
Version *F* value (*η* ^2^)	5.04* (0.005)	22.04** (0.02)	8.25** (0.01)	3.58† (0.003)	6.17* (0.01)
Interaction (*η* ^2^)	4.96** (0.01)	2.67† (0.004)	9.09** (0.01)	2.37 (0.004)	6.37** (0.01)
*Females*					
Group *F* value (*η* ^2^)	1723.57** (0.55)	1753.16** (0.52)	1159.19** (0.43)	308.60** (0.15)	1493.68** (0.50)
Version *F* value (*η* ^2^)	11.00** (0.01)	11.20** (0.01)	0.44 (0.001)	6.79* (0.01)	0.84 (0.001)
Interaction (*η* ^2^)	9.39** (0.02)	6.37** (0.01)	8.43** (0.01)	4.43* (0.01)	9.42** (0.02)

† = *P* < 0.05; * = *P* < 0.01; ** = *P* < 0.001.

There was a main effect of group and version for all subscales and an interaction on all subscales except for the attention to detail subscale in males. To elucidate these findings and to test the hypothesis that siblings would score higher than control individuals, Bonferroni post hoc tests were calculated for all comparisons. For males, the only subscale where ASC > siblings > controls after correcting for multiple comparisons was the Communication subscale; siblings and controls did not statistically differ from each other (though both differed from ASC individuals) on all other subscales. For females, however, all groups differed such that ASC > siblings > controls for all subscales except for the Imagination subscale (where siblings did not significantly differ from controls). In addition, with regard to version, for both males and females, subscale scores for the AQ consistently differed from AQ‐adolescent and AQ‐child scores (though parent‐report versions did not statistically differ from each other except for in females on the Communication subscale). Taken as a group, parent‐report subscales were significantly lower than self‐report estimates.

## Discussion

People with ASC on average score higher on the AQ than individuals with no clinical diagnosis [Auyeung et al., 2008; Baron‐Cohen et al., 2006, 2001]. A systematic review summarized that this difference can be observed whether or not the non‐autistic individuals have a family history of autism [Ruzich et al., 2015a]. The current study confirms these findings, as in all cases the ASC group scored higher on the AQ, and AQ subscales, than siblings or controls. However, the AQ also highlights the spectrum nature of autism, such that when examining group means (as in Table 2), a continuum is seen clearly extending from the clinical range through to controls. This study supports the hypothesis that autistic traits are elevated in parents and siblings of individuals with ASC compared to individuals with no family history of autism. The current study found a number of patterns indicating that siblings differ in interesting ways from typical controls with no personal or family history of ASC.

Examining total AQ scores, we predicted that individuals with ASC would score highest, followed by siblings, and then by controls. We found this pattern in females, where individuals with autism scored significantly higher than siblings who in turn scored significantly higher than controls, irrespective of which version of the AQ was used. For males, however, this trend was only evident on the adult (self‐report) version of the AQ, while for the Adolescent and Child (parent‐report) versions there was no difference in autistic traits as measured by the AQ between siblings and controls. This leads to the conclusion that self‐report versions for males have a discriminatory ability that is lacked by the parent‐report versions. A closer examination of subscale scores found that, for males, the only subscale where siblings differed from controls was the Communication subscale, suggesting that language and communication is a vulnerable area for autistic traits in males. Subscale scores for females, on the other hand, showed that siblings fell between individuals with ASC and control participants on all subscales, with the exception of the Imagination subscale (when AQ version is taken into account, there may also be some exceptions for parent‐reported Attention to Detail and Attention Switching subscales). Thus it appears that participant sex and AQ version are as important as sibling status when predicting AQ scores.

With regard to sex, we found that, like controls, there was a significant difference between males and females for child siblings, while no difference was observed for adolescent or adult siblings; it is possible that, as sex differences tend to be small in effect size, this is related to sample size and power. Conversely, this observation may have arisen as a result of differences in how the study populations were ascertained, or even in the measures themselves and how they were used. Future longitudinal research could examine how sex differences in AQ scores of siblings of people with ASC might change over time. When considered as distinct groups, we found that male and female siblings only differ for AQ‐Child scores, while AQ‐Adolescent and self‐report AQ scores are comparable for males and females. This leads to the conclusion that sexual dimorphism of autistic traits in siblings, unlike in controls, is reduced, though not to the same extent as it is for individuals with ASC. One interpretation is that in both people with ASC and in their siblings, typical sexual dimorphism is attenuated over the developmental time‐course [Baron‐Cohen et al., 2014], such that there are sex differences in siblings early in childhood, but as time progresses, these differences diminish to the point of non‐existence. We conclude that males and female siblings should be studied separately and that developmental processes that typically give rise to cognitive sexual dimorphism may be affected in ASC and their first‐degree relatives.

Version too needs to be taken into account: when examining the effect of version on AQ scores, we found differences between self‐report and parent‐report formats. The AQ, AQ‐child, and AQ‐adolescent were developed to measure autistic traits across the lifespan, and therefore they use similar questions and are frequently used by researchers and clinicians in the same manner. Further, ASC and autistic traits demonstrate stability over the lifespan [Holmboe et al., 2014; Robinson et al., 2011]. However, no study has, until now, thoroughly examined the effect of parent‐report versus self‐report on the AQ. The present study found that, for the most part, the AQ‐adolescent and AQ‐child are comparable measures that vary only on item age‐appropriateness. This bolsters the original aim of the AQ‐Child, which “was adapted from the adult and adolescent versions of the AQ, and items that were not age‐appropriate in the adult questionnaires were revised accordingly. Items in the AQ‐Child were kept as close to the AQ‐adult and AQ‐adolescent as possible, with most questions aimed at the same behaviors. Items were worded to produce an approximately equal agree/disagree response in order to avoid a response bias” [Auyeung et al., 2008]. However, we recommend that caution should be exercised when comparing self‐ and parent‐report scores, as we found that scores on the parent‐report AQ versions tend to be distributed differently than scores on the self‐report AQ.

It is possible that the current findings can also be interpreted to some extent by considering how individuals perceive themselves, how parents conceptualize their children, and how society influences behavior. For parent‐report versions of the AQ, male siblings and male controls did not differ in total score, suggesting that parents may rate their child with ASC as having many autistic traits and their male child without ASC as indistinguishable from a typically developing male child. Conversely, parents of girls with a sibling with ASC positively identify more autistic traits in their child. It may be that, in male children and adolescents, this may be due to a contrast effect whereby parents create a dichotomy between their male child with ASC and their male child without ASC. Further investigation is necessary to fully disentangle the relationship between sex and sibling pairs and to better understand why this pattern would hold for male but not female children.

### Limitations

There are limitations to this study that should be acknowledged. First, we are limited by the reliability of the entries submitted to the research database. Effort has been made to ensure diagnostic information and sibling status is accurate, but like the AQ responses themselves, these details are self‐ or parent‐reported and thus have not been verified by a researcher on our team, although diagnosis was made by an appropriate professional (psychiatrist or clinical psychiatrist) in a recognized clinic. Beyond accurate diagnostic status of the individual, we were unable to verify the extended family history for each participant who took part in the study. In addition, there is some uncertainty in the interaction between respondent sibling sex (male or female) and sex of individual with ASC. Four possible combinations can exist: a male sibling of a male individual with ASC, a female sibling of a male individual with ASC, a male sibling of a female individual with ASC, and a female sibling of a female individual with ASC. It might be expected that this factor, particularly with respect to the parent‐report versions of the AQ, would have an effect on AQ scores. Further, birth order is likely to be relevant as well. However, the current study lacked the statistical power to make this comparison, especially in light of the fact that the male to female ratio for autism across the spectrum is 4:1 [Wing, 1981], making it difficult to recruit sufficient numbers of females.

Second, the current study only focused on basic descriptive statistics such as means, ranges, and standard deviations of scores. A closer examination of the reported AQ score distributions (Fig. 1) indicates there may be a bimodal distribution of scores for siblings, leading to the hypothesis that distinct subgroups exist for the sibling group with varying levels of autistic traits. In a separate study, we report research that tests whether clear subgroups exist, and to what extent some or all of these subgroups have a broader autism phenotype [Ruzich et al., 2015b].

## Footnote

1 For further comparison of these and other measures, see Nishiyama et al. [2014].
